# Dental Jurisprudence

**Published:** 1914-10-15

**Authors:** 


DENTAL JURISPRUDENCE. This is the title of a new book by Elmer D. Brothers, Professor of Medical and Dental Jurisprudence in the University of Illinois and Lecturer in the John Marshall Law School of Chicago. The book is small, about 200 pages and is intended to epitomize laws governing or involved in the practice of dentistry. It is not technical, but is written in a style that makes it not only readable by the dentists, but the subjects treated are of such vital interest that every dentist will be greatly interested and profited by a careful study of its contents. Practically every subject that a dental practitioner is likely to be interested in is carefully discussed, and where litigation has occurred the cases are cited with reference notes. This feature will make the book invaluable for State Board Officers who have to prosecute illegal practitioners, and for lawyers who may not be familiar with dental litigation, enabling them to find data which might apply to cases in which they are engaged. By far the most important use of the book will be to make the profession intelligent as its relations to the principles of law controlling business affairs. Dental students as well will find their rights and obligations clearly set forth, so that they may govern their actions legally. Of all the books relating to the subject we believe this the most satisfactory and we cordially recommend it to every professional man. It will make any dentist who reads it carefully a better business man as well as a law abiding citizen. It is published by The C. V. Mosby Co., St. Louis, at two dollars.

